# Great Expectations: A Qualitative Analysis of the Factors That Influence Affective Forecasts for Exercise

**DOI:** 10.3390/ijerph17020551

**Published:** 2020-01-15

**Authors:** Amanda J. Calder, Elaine A. Hargreaves, Ken Hodge

**Affiliations:** 1 National Institute for Health Innovation, University of Auckland, Auckland 1142, New Zealand; 2 School of Physical Education, Sport and Exercise Sciences, University of Otago, Otago 9016, New Zealand; elaine.hargreaves@otago.ac.nz (E.A.H.); ken.hodge@otago.ac.nz (K.H.)

**Keywords:** motivation, cognitive appraisal, affective responses, physical activity, behavior change

## Abstract

The extent to which people expect to feel pleasure during exercise is proposed to influence an individual’s decision to be active. In order to identify the factors that shape this affective forecast for exercise, this study explored what people think about when creating their affective forecast for exercise. Thirty-one inactive participants provided an affective forecast for a moderate intensity exercise session using the global affective forecast questionnaire. Immediately after, they were asked a series of questions to verbally explain what they were thinking about in order to generate their forecast. Thematic analysis identified four themes relating to the exercise intensity, the exercise outcomes, the exercise environment, and the enjoyment of exercise that influenced affective forecast creation. Exercise practitioners should design strategies to manipulate these factors, and structure exercise environments to support a positive affective forecast and better motivate exercise participation.

## 1. Introduction

Physical inactivity levels are increasing and cost nearly 3.2 million people their lives each year [1]. As a means of increasing motivation to be active, research has investigated the role of affect variables [2,3]. Positive affect, a key component of psychological well-being, has been associated with exercise [4,5]. One specific affect variable is the individual’s affective forecast. This is the valenced prediction people make for how they think they will feel in response to a future stimulus, be it a behavior, event, or situation [6,7,8]. These affective forecasts can elicit action tendencies to approach or avoid certain behaviors depending on whether they are framed as positive or negative, respectively [9]. Affective forecasts have been proposed to influence the decision to be active [10,11] and found to be associated with exercise intentions and behavior [12,13]. Consequently, ensuring individuals forecast positive affective responses during their next exercise experience is important for motivation. Yet, the factors that influence the creation of affective forecasts in the exercise context have not been investigated.

Williams et al. [10] proposed that if an individual makes a positive affective forecast for exercise, then they would be more likely to engage in exercise, and vice versa for a negative affective forecast. As discussed by Williams [14], these proposals are based on the hedonic principle [15], which states that people aim to increase pleasurable experiences and minimize the occurrence of unpleasurable experiences. Outside the exercise context, research has demonstrated the influence of affective forecasts on decisions. For example, gamblers bet on the option they forecasted would result in the most positive affective response [16]. A meta-analysis showed anticipated affective reactions were significantly correlated with health behaviors, including physical activity and exercise, and significantly predicted intentions to participate in those health behaviors, which included exercising regularly [12]. In an experimental study, Kwan et al. [13] manipulated affective forecasts for exercise to be more positive or negative. Significant associations were found between those manipulated affective forecasts and intentions to exercise. Interestingly, there was no association between those affective forecasts and exercise behavior, but Kwan et al. [13] explained that the manipulation might not have been strong enough to create an affective forecast that would influence exercise behavior.

Research outside of the exercise context has described factors that have influenced the creation of affective forecasts. Specifically, affective state at the time of making the forecast [17], the timing of the event relative to when the forecast is made [18], not having enough information about the behavior [19], previous experience of the event being forecasted [20], and the personality traits of the individual [21] have all influenced affective forecasts. These studies demonstrated that these factors changed the valence of affective forecast and showed that previous experience changed the affective forecast. For instance, participants who did not have experience using public transport forecast less satisfaction for their first experience compared to their second experience [20]. This research indicates that there are multiple factors that influence affective forecasts and it is likely that a similar situation would exist in the exercise context.

Very little research has investigated the factors that influence affective forecast for exercise. Loehr and Baldwin [22] found that active people forecasted more enjoyment for exercise than inactive but reported the same amount of enjoyment after the exercise, supporting the role of previous exercise experience in influencing an affective forecast. Ruby et al. [23] found that participants expected to enjoy exercise more when their favorite part of a workout happened first rather than last, suggesting the structure of the exercise workout influenced affective forecasts. Lastly, Zenko et al. [11] found that participant’s affective forecasts were more positive after having experienced increasingly positive affective responses during exercise, compared to a decrease in positive affective responses. This suggests that experiencing positive or negative affect during exercise influences the affective forecast for future exercise. This research suggests that people may create affective forecasts from affective memories. Indeed, explicit affective memory from exercise has been suggested as the predictor of affective forecasts [5]. Yet, affective forecasts can be created without having previous experience [20], indicating that other factors besides, or in addition to, those stemming from memory could influence affective forecasts.

The extent to which affective responses during exercise are positive or negative is determined by a combination of the interoceptive cues and cognitive appraisals the individual experiences as a result of the exercise intensity [4]. Research that investigated the cognitive appraisal element of how affective responses during exercise are created reported factors relating to pre-exercise affective state (e.g., having low levels of motivation) [24], outcome expectations (e.g., health benefits, enjoyment, sense of achievement) [24,25], self-efficacy [24,26,27], attentional focus [24,28], and perceptions of physiological state [24,29]. Given that affective responses experienced during exercise and affective forecasts for exercise are related [11], it may be that similar factors influence the creation of affective forecast. No previous research has explored affective forecasts for exercise using qualitative methods. Consequently, the purpose of this study is to gain a comprehensive picture of the factors influencing the creation of an affective forecast for exercise.

## 2. Materials and Methods

### 2.1. Design

A longitudinal qualitative study was conducted employing descriptive phenomenology [30,31,32], which explores the meaning behind experiences and allows people to communicate their feelings, attitudes, and beliefs [33,34]. Participants were interviewed four times over a period of three months after completing one 30-min exercise session in a laboratory.

### 2.2. Participants

Twenty-nine female and two male volunteers (*M* = 38, *SD* = 9) were recruited from the local community through flyers placed in community areas (e.g., supermarket notice boards), emails sent to University staff members, advertising placed in the local newspaper, and on the radio. The ethnicity of the participants was 55% New Zealand European, 13% New Zealand Māori, 26% Other European, and 6% Other. Participants were eligible if they were aged between 18–60 years old, self-reported they were healthy, and participated in less than 150 min of physical activity per week. Excluded from the study were any participants who reported a condition or illness that would be exacerbated by exercise or reported taking any medications that influenced heart rate (e.g., beta blockers). The University ethics committee granted ethical approval (code: 15/087).

### 2.3. Procedure

#### 2.3.1. Exercise Session

Upon arrival at the laboratory, participants were told that they would complete a 30-min walk on the treadmill, 10 min of which would be a warm-up, and 20 min would be at a moderate intensity. Participants then began their exercise session, walking at 3 km·h^−1^ at a 2% gradient. Over the warm-up, the speed was gradually increased so that by the end of 10 min the participant was walking at an intensity corresponding to moderate intensity. After the 30-min of exercise and a subsequent two-min cool down, the participants were seated. Fifteen minutes after exercising, participants were asked to report their affective forecast for a future exercise session on the global affective forecast scale (GAF, [35]). Participants were asked, “what number best represents the overall amount of pleasantness or unpleasantness that you predict you will experience during the next 30-min moderate intensity exercise session you will complete, if negative ten is very unpleasant, zero is neutral, and positive ten is very pleasant?”. Participants responded verbally after viewing the scale which had written anchors at +10 (very pleasant), 0 (neutral), and −10 (very unpleasant) and 1-point increments. Participants were asked to provide this score to facilitate their ability to explain the factors that influenced why they chose that particular number in the subsequent interview.

#### 2.3.2. Semi-Structured Interview Procedure

After reporting their affective forecast, participants took part in a semi-structured interview with questions designed to elicit information about what influenced their reporting of the particular affective forecast score. We took guidance from qualitative work investigating affective responses to exercise [24,25,36] when developing the interview questions. Firstly, participants were asked, “What things are you thinking about to predict a (specific number given on the GAF scale)?” which invited participants to talk about anything that contributed to their response. Participants were then asked follow-up questions, if necessary, to expand on or clarify their answers. For example, “you say you are thinking of (satisfaction), what do you mean by (satisfaction)?” or “when you say you are thinking of (last time), what specifically about (last time) are you thinking about?”. The semi-structured interview was concluded when the participants had no more to say or when asked, “is there anything else that you are thinking of to predict feeling (number on the scale)?” and they answered there was not. The conversation was recorded on a Dictaphone (IC Recorder, ICD-PX470, Sony, Tokyo, Japan).

#### 2.3.3. Follow-Up

All 31 participants were followed up and contacted by telephone at a prearranged time seven days, one month (30 days, +/− 3 days), and three months (90 days, +/− 3 days) after the exercise session and asked to respond to the affective forecast question again to look at change over time. No participants dropped out of the study. Participants were reminded that they were on speakerphone and that the entire call was being recorded on the Dictaphone. They were then asked the same semi-structured interview questions as before to elicit information relating to what was influencing their chosen affective forecast response. Semi-structured interviews transition well from face-to-face interviews to telephone follow-up phone calls and enabled a more pragmatic approach to data collection [37,38]. Responses were repeated back to the participants during the interview, after which they were asked to verbally confirm their results.

### 2.4. Data Analysis

The semi-structured interviews were transcribed verbatim into separate (approximately) five-page documents for each of the 31 participants. The exploratory nature of the research question meant it was not based on pre-existing theory and themes were not pre-determined. Thus, to understand the underlying meanings and patterns in the data, the transcribed interviews were subjected to an inductive thematic analysis [39,40].

The thematic analysis was undertaken following the six-phase process described by Braun and Clarke [39]. (1) To ensure familiarization with the data, the interviewer re-read the transcripts in their entirety whilst listening to the audio-recording. Any initial ideas relating to the factors that influenced the creation of the affective forecast were noted on the transcripts. All quotes that pertained to what factors the participants were thinking about when creating their affective forecast were highlighted and placed in a separate word document with the participant number and the follow-up time point attached in a table. (2) Each quote was reduced to a few key words to create a code that summarized the quote. Where possible, words from the quote were used in the code, which was kept alongside the initial quotes in the table to maintain context. (3) Codes with similar patterns or meanings within the data were grouped together to identify a common theme that described the overall essence of those grouped codes, creating the first-order themes. (4) Each of the potential first-order themes were reviewed to ensure that each theme was distinct. Broader similarities identified in those first-order themes were grouped and labeled (second-order themes). The second-order themes were analyzed for distinctiveness, and any broad similarities were identified, grouped, and labeled (third-order themes). (5) Each of the first-, second-, and third-order themes were defined and given a name based on the general concept to which that theme pertained. (6) Data were reported to the other authors for triangulation and verification of the analysis to minimize investigator bias in the interpretations made [41]. The results were presented with example quotes to highlight the themes. Any unnecessary filler words (e.g., um) were removed and replaced with an ellipsis. Any words that were ambiguous (e.g., it, that) were replaced with the concrete noun in square brackets (e.g., [exercise]). Each of the revealed themes were considered important without calculating the number of times each theme was mentioned and were not considered to have any level of importance relative to each other [42]. To maintain context and highlight any subtle differences between the time points, the analysis was conducted separately for each time point, and each quote has the participant who provided the quote and the time it was recorded noted next to it (e.g., Participant 1, 15 min). During the analysis process, it was recognized that data saturation had occurred, as no new themes were emerging [43].

### 2.5. Research Rigor

The following criteria were employed to maintain research rigor and trustworthiness [44,45]. As described in the introduction, the research was a topic worthy of being researched with both appropriate data analysis and a full description of procedures. Data were collected using the inductive interpretivist analysis process, which is often utilized in sport and exercise psychology [46]. The lead researcher (AC) reported the processes honestly for research sincerity. To ensure research credibility, data from different time-points were initially kept separate. The participants reported a range of ethnicities, and both males and females volunteered, providing multiple and variable voices to the topic. The narrative context of the data was maintained through rich descriptions from the participants for the research to have transferability and resonance (the potential to influence many lives). This research provided a significant contribution given that no study has explored what factors create affective forecasts for exercise. The research achieved procedural ethics through the ethical approval granted by the University ethics committee and achieved situational and relational ethics through consultation with the Māori research committee. Finally, the research had meaningful coherence by using data analysis methods that were appropriate for the overall methodological approach of the current study, the research question of the current study, and the field in which the current study resides; sport and exercise psychology [40,46].

## 3. Results

Similar first- and second-order themes were identified during the analysis of the data collected at 15 min, one week, one, and three months following the initial exercise session. Consequently, the results were presented with all data combined. Four third-order themes were identified: interpretations of the exercise intensity, the outcomes of exercise, the exercise context, and enjoyment from exercise. First-, second-, and third-order themes are shown in Table 1.

### 3.1. Interpretations of the Exercise Intensity

This theme explains the individuals’ understanding of the physical demands of the exercise, and their capacity to meet those physical demands. Three first-order themes were identified as important to its creation: (1) the intensity of previous exercise, (2) the future exercise intensity, and (3) perceived ability to exercise at the exercise intensity.

Participants described thinking about the previous exercise intensity as manageable, comfortable, not too hard, and enjoyable, and this influenced their affective forecast. As participant 1 explains, their affective forecast was influenced by the intensity of the laboratory-based exercise session they had just completed,

If [the exercise] is at the same intensity and about the same length of time [as the treadmill session] as I said I don’t want to go on any longer...so that [exercise session] was pleasant because I was moving, I was walking, it was a pace I could manage, and…the timing, as I said the length of time was good for me so that was all positive, so if I had to do it again I would feel good about it.

Participants discussed thinking about whether the intensity of the future exercise would be manageable, beneficial, controllable, a strain, hard, and intense, which influenced their affective forecasts. For example, participant 10 stated, “I think it is going to be core [exercise class] tonight so…I quite like core because it is not too intense and sweat[y]” (three months), while participant 24 explained, “I think that the next half hour of work that I do will probably be pretty hard” (one month).

Participants’ perceptions of their ability to do the exercise depended on the intensity of the future exercise. In explaining a more positive affective forecast, participants described that they would feel confident about completing the exercise and knowing “just that I can do [exercise]” (participant 14, 15 min). Participants also described feeling an improvement in their abilities, for example, “[I am feeling] really good about being able to walk on the treadmill right through, previously I would have to stop…but now I can do until twenty minutes without stopping without…needing a water or feeling tired” (participant 25, one month). One participant explained that they were worried about whether they could complete their next exercise session due to pain from previous exercise and this negatively influenced their affective forecast;

I think [exercise] will be a bit painful because I have got sore calf muscles so I think it will be quite unpleasant, however that won’t stop me…well I am wondering about this sore calf muscle and the strain in my back side, and all the things that have told me that I have played tennis for two hours at a higher level than I should have done, so just and…I am worried and I am wondering and thinking “ooo can I do it” and “is it going to be terrible or not” (participant 1, three months).

### 3.2. The Outcomes of Exercise

This theme explained the corporeal and cognitive consequences individuals expected to obtain from participating in exercise and was underpinned by (1) experiencing benefits from exercise, and (2) the physiological sensations from exercising.

#### 3.2.1. Experiencing Benefits from Exercise

Affective forecasts were influenced by whether participants would feel better and feel mindful during exercise. For example, “I think…[exercise] works as meditation because…I am mindful of what I am doing, I am away [from] my worries so I think that is good” (participant 23, one week). 

Participants thought about how they would feel immediately after exercise when creating an affective forecast, even though participants were specifically asked to explain their affective forecast for during exercise. For example, when asked to explain why hard exercise was positive, participant 10 explained, “even though yeah [the exercise] might be hard but you are like ‘come on, almost there’ and then you finish and you feel good” (15 min). Similarly, participants described their affective forecast was influenced by the expectation that they would feel calm, productive, energetic, relaxed and fulfilled immediately after exercising.

Knowing they would feel a sense of accomplishment from doing some exercise influenced the forecast. For example, participants described, “feeling the satisfaction of doing some activity” (participant 31, three months), feeling as though exercise is a worthwhile experience, and “just knowing that I have done a bit of exercise, it always makes you feel better” (participant 4, one month).

One specific outcome described by participants was that exercise gave them a chance to get away, get out, be alone, do something for themselves, and take time for themselves, which influenced their affective forecast in a positive way. For example,

well it is time out, so I am not…doing stuff for everyone else. So it gives me time away from kids, from other things that I need to be dealing with. It is just time for me so I am doing something for me that I enjoy, and it’s looking after myself…It is like when I go out and I am being active, whether it is going for a walk or if I am on a rowing machine or whatever, it is…time that I am spending for myself not anyone else (participant 2, one week).

#### 3.2.2. Physiological Sensations from Exercising

Affective forecasts were positively influenced by participants expecting to feel warmed up, puffed, loosened up, feel their muscles working, their blood pumping, and their body moving. For example, “yeah just my body feeling like it’s having a bit of exercise, getting a bit of a workout, feeling my muscles working...getting some air into my lungs you know, just those physical sensations of actually exercising” (participant 15, one week).

Participant’s beliefs about getting longer-term health benefits from exercise also influenced their affective forecast;

the fact that doing exercise is a step towards hopefully being in better shape…I also kind of get the positive of like…this is good for my back, I am sure this is improving my overall situation, not just like the weight thing or fitness levels thing…this is something that will hopefully make me feel better when I am sitting at my desk tomorrow (participant 29, 15 min).

If a participant had not experienced any negative outcomes whilst exercising (e.g., pain), they hoped that the next exercise experience would also be pain free, which positively influenced their affective forecasts.

Affective forecasts were negatively influenced by participant’s expectations that they would feel sore, tired, or that they would feel pain during, or after, exercising. For example, when explaining why they were thinking about being in pain during exercise, participant 17 said, “last week I ended up feeling the pain in my legs…I didn’t feel it whilst I was doing it, just near the end, but like the next day I did feel the pain” (one week). Participants expressed hope that these negative outcomes would not occur again, however, the possibility they could influenced the creation of their affective forecasts. For example, participant 22 explained, “…yeah as well I haven’t been [in pain] as much the last few walks, so I hope that that might indicate that I won’t be next time either, but you never quite know” (one month).

### 3.3. The Exercise Context

This theme explained the physical exercise setting and the participant’s opinions about exercise. Underpinning this theme were two second-order themes: the exercise environment, and motivations to exercise.

#### 3.3.1. The Exercise Environment

Where participants planned to exercise influenced their affective forecasts. Participants who were going to exercise in their preferred location, either outdoors, at home, or in a gym, described a positive affective forecast. For example, one participant who planned to exercise at home explained that they felt they were in a safe environment, while participant 5 explained, “[I] will be doing exercise for pleasurable reasons, getting out and enjoying the day…fresh air” (one week). Participants who were going to exercise in a location they did not like, whether that was outdoors, in the gym, or at home, described a more negative affective forecast. For instance, one participant explained that their affective forecast was negatively influenced by exercising outdoors, because they would not feel comfortable exercising in public. Another stated,

I get frustrated with the whole, go and get changed, and wipe the machines, go back. So I do it at the end of the day… [I] can’t be bothered, I don’t like [the gym] so that’s why [my forecast is] not a massive number (participant 13, 15 min).

Participants explained that exercising with someone was pleasant, nice, enjoyable, and fun, which positively influenced their affective forecast. For example, participant 7 stated, “especially if you have got someone with you it’s actually quite an enjoyable experience, it’s having someone there it’s sharing the experience” (15 min).

#### 3.3.2. Motivations to Exercise

Participants explained that wanting to exercise, feeling enthusiastic, and a need to do exercise positively influenced their affective forecast. For example, “I am looking forward to doing more [exercise] so I am optimistic and so looking forward to it” (participant 8, one month), and “[I’m] just enthusiastic about [doing exercise] I suppose” (participant 18, one month), and participant 5 explained,

because I am I’m stuck in front of a computer all day so that’s why I find it very important to get outside and get moving, just knowing that [exercise is] something that I need to do because I don’t get the choice during my work hours (one week).

Affective forecasts were negatively influenced by participants’ expectation that they do not like exercising. For example, participant 6 stated, “well I am thinking of neutral [on the GAF scale] because I don’t like exercise, it is not something that I willingly do” (one week).

### 3.4. Enjoyment from Exercise

This theme explains the enjoyment that individuals associated with exercise specifically relating to the enjoyment of the previous exercise experience, future exercise, and of exercise in general.

Affective forecast was influenced by how much participants enjoyed their most recent exercise experience. This was either the laboratory-based exercise session, for example, “Just because I enjoyed today so much” (participant 5, 15 min), or another form of recently completed exercise, “I enjoyed the aqua jogging so I know [the exercise is] going to be fun” (participant 12, one week), or both. For example, participant 26 stated, “I quite enjoyed the exercise I did with the treadmill, and I also have been enjoying getting out in the garden and just doing a bit more and feeling it, basically feeling the work” (one week). Affective forecast was positively influenced by how much participants expected to enjoy the exercise they planned to do.

Affective forecasts were influenced by how much participants enjoy exercise. For example, participant 19 stated, “just cause I enjoy exercise…I just like the physical movement of it… It’s just nice to be doing something…” (one week). Participants also described enjoying the challenge of exercise, and the exercise experience; “the fact that I enjoy [exercise] and yeah…you know just the entire experience is something I enjoy” (participant 2, one month).

## 4. Discussion

The purpose of this study was to explore the factors that influence the creation of affective forecasts for exercise. Results showed that how people think they are going to feel during exercise is influenced by the physical demands of the exercise intensity and their ability to meet those demands; the physiological and psychological benefits they expect to obtain from the exercise; the context in which exercise will take place; and the enjoyment associated with the exercise. Each theme reflected expectations gained from previous exercise experiences (e.g., the sensations of exercise, enjoyment), or what would occur in the future exercise session (e.g., the intensity of the exercise, where they would exercise). The participant’s interpretation of these factors created more positive or less positive affective forecasts.

The intensity of previous and/or future exercise sessions were key influences on the creation of an affective forecast. Exercise intensity is a strong determinant of the affective response to exercise [4] and how people interpret their exercise intensity plays a role in the cognitive appraisal process that informs affective responses to exercise [24]. The present study demonstrated that exercise intensity also influences affective forecasts. When anticipating how exercise will make them feel, participants recalled how manageable and/or intense previous exercise experiences were and evaluated their future chosen intensity for similar attributes. Encompassed within this explanation were participant’s reflections of their confidence or ability to manage exercise at the anticipated intensity. This appraisal of self-efficacy has also been shown to influence the affective responses to exercise [47,48].

The influence of exercise outcomes shared similarities to research that found factors relating to the immediate outcomes of exercise [25], the sense of achievement, and long-term outcomes [24] influenced affective responses during exercise. Individuals in the present study thought about the physiological and psychological outcomes they would experience from exercise when creating their affective forecasts. The physical and mental benefits from exercise are well-documented [1,49] and when participants were expecting to feel these benefits, their affective forecast for exercise was more positive. Given the predictive relation between affective responses and affective forecasts demonstrated in exercise [11], it was not unexpected that the outcomes from exercise were identified as an influence on affective forecasts.

Interestingly, participants discussed how they would feel after exercise as an influence on their affective forecast during exercise. According to the peak and end rule, people recall the most intense affective response and the affect experienced at the end of an event to create an affective memory [50]. Support for this premise has been found in the exercise context [51]. Given that previous experience influences the creation of affective forecast [20], and affective memory is associated with affective forecast [11], this may explain why post-exercise affect plays a role in creating the overall affective forecast. There are differing suggestions for when the ‘end’ of an exercise session is; on immediate cessation of exercise, or on the normalization of physiological responses [51]. The complexity of when the end of an exercise session occurs could explain why participants mentioned how they would feel immediately after exercising as influencing their affective forecast. However, an alternative explanation may be that participants misunderstood the question and were providing explanations for their post-exercise affective forecast and not their during-exercise affective forecast. Future research should investigate the role of post-exercise affect and the wider tenets of the peak and end rule in affective forecast generation.

Affective forecasts for exercise were influenced by the environmental context and motivational circumstances that encompassed the exercise experience. People have retrospectively described exercise in a natural setting as fun, that they were feeling good, and that the surroundings were beautiful [52], which shared similarities with how participants in our study explained exercising outdoors would positively influence their affective forecast. Conversely, the exercise setting negatively influenced affective forecasts for some participants. Similarly, exercising in company has positively influenced affective responses [53] and affective forecasts in this study. For some participants, however, the presence of others negatively influenced their affective forecast. These results raise the question of why people would choose to exercise in a situation that they know will not make them feel pleasant, or that makes them uncomfortable. There could be barriers (e.g., financial, location) limiting the choice of exercise location, but we do not have data to do more than speculate. However, it does point to an interesting health promotion strategy to facilitate positive affective responses to exercise: Exercise in a place or with people that will make you feel good.

Participants also explained that their desire to exercise and whether they liked exercise or not informed their affective forecast. In their affective-reflective theory of physical activity and exercise (ART), Brand and Ekkekakis [54] discuss the role of automatic (implicit) and reflective (explicit) processes in assigning an affective valence to exercise, which then drives exercise decisions. The extent to which participants’ motivational state is positive or negative could activate the individual’s automatic association to exercise which feeds into the affect system. Further research using ART as the guiding framework will elucidate these relationships.

The expected and remembered enjoyment of exercise influenced the creation of the affective forecasts. This result extended research demonstrating that enjoyment experienced during exercise influenced the creation of affective responses during exercise [24,52]. Enjoyment is an emotion that is encompassed within the concept of positive affect, whereas emotions such as sadness and disgust are comprised of negative affect [4]. Therefore, if people experience positive affect as enjoyment when exercising, then affective forecasts for future exercise will be positively influenced by that enjoyment.

There were references to previous exercise experiences within all four themes. For example, participants discussed previous exercise intensity, previously experienced outcomes from exercise, previous exercise settings, and enjoyment of previous exercise sessions as influencing the creation of their affective forecast. Research has demonstrated a correlation between affective memories and affective forecasts [11], and our results extend this research by explaining the specific factors from exercise experiences that were remembered and how the appraisal of those factors as positive or negative influences how participants expected to feel when they exercised again.

As with all studies, there were some limitations. One limitation is the extent to which participants were able to verbalize the cognitive factors influencing their affective forecasts and therefore provide a full account of their experiences. Nisbett and Wilson [55] doubt that people can accurately articulate the cognitive processes that occur in reaction to stimuli. They argued that people rely on heuristics and judgments when they explain their thought processes, although they conceded that some accounts must be accurate [55]. In the present study, there might have been subconscious or implicit thoughts regarding exercise that could not be identified. For instance, a strong relation has been shown between implicit attitudes and exercise [56], which could also influence affective forecast. Consequently, there might be other factors involved in the creation of affective forecasts for exercise that could not be identified in this study. Furthermore, all participants in this study had experienced exercise through the moderate intensity session prior to making their affective forecast. Given that people underestimate how good they will feel during exercise [22], there might be other factors involved in the creation of an affective forecast for people who are starting out with exercise and who do not have any prior experience that have not been identified in this study. Additionally, given the affective forecast question was repeated four times over three months, participants may have simply mentioned factors that they remembered describing previously regardless of whether their affective forecast was influenced by that same factor.

The way in which the affective forecast question was framed might have influenced the data collected. Firstly, participants were asked to forecast their affect for their next 30-min of moderate intensity exercise. Given the prompt to think about a specific intensity of exercise, this may have led to the emergence of the theme relating to the intensity of the upcoming exercise session. Secondly, at the time of asking the question, some individuals did not know what form their next exercise session would take and had to imagine their possible future exercise options. Consequently, their explanations for their affective forecasts were more speculative and general compared to those participants who knew what exercise they were going to do and described their forecast for that specific exercise session. Although these results highlighted the complex creation of affective forecasts for unplanned exercise as well as planned exercise, future research should consider the content of the affective forecast question used to ascertain factors that may be specific to affective forecasts for a planned exercise session. One final note is the substantially higher number of females to males. Although research has not shown any evidence of gender influencing affective forecasts [57], it would be pertinent to confirm in future research that these findings are also relevant to men.

## 5. Conclusions

When creating an affective forecast for exercise (an expectation of the extent to which exercise will feel pleasurable, or not), individuals think about the intensity of the exercise, the direct physical and mental health outcomes expected from the exercise, the social and physical context in which the exercise takes place, and the enjoyment that they associated with exercise. This appraisal is informed by memories gained from previous exercise experience as well as anticipation of the future exercise components. A positive affective forecast is proposed to influence the decision to exercise or not. These results provide those involved in physical activity promotion, and indeed, the individual exerciser, the basis for developing strategies to create exercise environments that facilitate positive affective forecasts for exercise.

## Figures and Tables

**Table 1:** Themes illustrating the factors that influenced the creation of an affective forecast for exercise.

First Order Theme	Second Order Theme	Third Order Theme
The Intensity of Previous Exercise	Interpretations of the Exercise Intensity	
The Future Exercise Intensity		
Perception of Ability to do the Exercise Intensity		
Positive Psychological Outcomes	Experiencing Benefits from Exercise	The Outcomes of Exercise
Sense of Accomplishment from doing Exercise		
Exercise as a form of Me Time		
Positive Physiological Sensations	Physiological Sensations from Exercising	
Negative Physiological Sensations		
Exercise Location	The Exercise Environment	The Exercise Context
Sharing the Exercise Experience with Someone		
Desire to Exercise	Motivations to Exercise	
Negative Attitudes towards Exercise		
Enjoyment of the Previous Exercise Experience	Enjoyment from Exercising	
Enjoyment of the Future Exercise Session		
Enjoyment of Exercise in General		
